# The Hemoglobin, Albumin, Lymphocyte, and Platelet Score as a Prognostic Indicator for Dogs with Congestive Heart Failure Secondary to Myxomatous Mitral Valve Disease

**DOI:** 10.3390/vetsci12090908

**Published:** 2025-09-18

**Authors:** Jayeon Park, Yeon Chae, Sungjae Lee, Yoonhoi Koo, Hakhyun Kim, Byeong-Teck Kang, Taesik Yun

**Affiliations:** 1 Laboratory of Veterinary Internal Medicine, College of Veterinary Medicine, Chungbuk National University, Cheongju 28644, Republic of Korea; 2 College of Veterinary Medicine, Kyungpook National University, Daegu 41566, Republic of Korea

**Keywords:** CHF, dog, HALP score, MMVD, prognosis, short-term mortality

## Abstract

Reliable prognostic indicators for congestive heart failure (CHF) secondary to myxomatous mitral valve disease (MMVD) in dogs are limited. The hemoglobin, albumin, lymphocyte, and platelet (HALP) score, a prognostic marker in humans, has not been evaluated in veterinary medicine. This study aimed to assess the HALP score’s utility for predicting short-term mortality in dogs with CHF due to MMVD. This retrospective study included 54 small-breed dogs. The HALP score was calculated as: hemoglobin (g/L) × albumin (g/L) × lymphocytes (/L)/platelets (/L). Six-, nine-month, and one-year mortality were assessed. The HALP score was significantly higher in survivor groups. Receiver operating characteristic (ROC) analysis showed good predictive accuracy for six- and nine-month mortality (area under the curve > 0.7). A cut-off of 11.13 for six-month mortality yielded 44.44% sensitivity and 94.44% specificity. Kaplan–Meier analysis confirmed that a higher HALP score was associated with significantly longer survival. The HALP score appears to be a valuable, novel prognostic indicator for short-term mortality in dogs with CHF due to MMVD.

## Simple Summary

Reliable prognostic indicators for canine congestive heart failure (CHF) due to myxomatous mitral valve disease (MMVD) are needed. We evaluated the hemoglobin, albumin, lymphocyte, and platelet (HALP) score’s utility for predicting short-term mortality in 54 dogs with MMVD-related CHF. In this retrospective study, the HALP score was significantly lower in non-survivors. It demonstrated good predictive accuracy for short-term mortality (area under the curve > 0.7) with high specificity. Kaplan–Meier analysis confirmed that a lower HALP score was associated with significantly shorter survival times. The HALP score is a valuable and accessible prognostic indicator for dogs with CHF secondary to MMVD.

## 1. Introduction

Myxomatous mitral valve disease (MMVD) is one of the most prevalent chronic acquired heart diseases in small-breed dogs [1]. MMVD is characterized by the progressive degeneration of the mitral valve, leading to mitral regurgitation. This process results in left atrial (LA) and ventricular dilation and increased LA pressure, ultimately progressing to congestive heart failure (CHF) [2].

The progression of MMVD from its early stages to CHF has been a focus of numerous veterinary studies. For instance, various predictive indicators have demonstrated utility in assessing MMVD severity and predicting progression to CHF. These include echocardiographic parameters, such as LA and left ventricular dimensions; cardiac biomarkers, like N-terminal pro-B-type natriuretic peptide and cardiac troponin I; and systemic inflammatory markers, such as the neutrophil-to-lymphocyte ratio [2,3,4,5,6,7,8,9,10].

Despite significant advances in identifying indicators that predict progression to CHF, markers for predicting survival time in dogs that have already developed CHF remain limited. For dogs with CHF secondary to MMVD, the median survival time is approximately 281 days, but it can range widely from 3 to 885 days [11]. This wide variability in survival time suggests the need for reliable prognostic indicators and makes it challenging to predict individual outcomes and tailor treatment plans effectively. Unlike markers that assess MMVD severity, survival predictors serve a distinct purpose by offering crucial insights into disease outcomes after the onset of CHF. Reliable prognostic indicators are essential for assisting veterinarians in clinical decision-making and for providing pet owners with accurate information about their pet’s condition and prognosis.

In human medicine, inflammatory and hematologic biomarkers have been extensively utilized to predict prognosis in various diseases [12,13,14,15]. Recently, the hemoglobin, albumin, lymphocyte, and platelet (HALP) score has received significant attention as a novel prognostic marker. This score, derived from four easily accessible blood parameters, reflects a patient’s nutritional status, systemic inflammation, and overall health status. The HALP score has been extensively studied in various human diseases, including cancers, pancreatitis, and acute ischemic stroke, where it has demonstrated value in predicting patient outcomes [12,13,14,15]. Notably, in patients with heart failure, the HALP score has been shown to predict short-term mortality [15,16]. However, the prognostic utility of the HALP score has not yet been investigated in veterinary medicine.

The proven value of the HALP score as a prognostic indicator in human heart failure suggests its potential for application in veterinary medicine. The current lack of reliable prognostic indicators for survival in dogs with CHF due to MMVD highlights the need for accessible and effective new tools.

This study aimed to evaluate the prognostic utility of the HALP score for predicting six-month, nine-month, and one-year mortality in dogs with CHF due to MMVD. By evaluating the relationship between HALP scores and survival times, this study sought to determine whether higher HALP scores are associated with better outcomes, as has been demonstrated in human studies. Furthermore, this study explored the potential of the HALP score as a practical and readily available tool to assist veterinarians in predicting prognosis and guiding treatment decisions for dogs with CHF.

## 2. Materials and Methods

### 2.1. Animals

This retrospective study reviewed the medical records of 54 small-breed dogs diagnosed with CHF secondary to MMVD at the Veterinary Teaching Hospital of Chungbuk National University from January 2010 to December 2024. A total of 18 out of 54 dogs were receiving medication for the management of stage B2. The treatment regimens were as follows: pimobendan monotherapy (*n* = 2), angiotensin-converting enzyme (ACE) inhibitor (enalapril) monotherapy (*n* = 3), and combination therapy with pimobendan and an ACE inhibitor (enalapril, *n* = 10; benazepril, *n* = 3).

Dogs were excluded from the study based on the following criteria: (1) presence of CHF caused by diseases other than MMVD; (2) diagnosis of a concurrent serious disease that could affect the HALP score, such as neoplasia, endocrine disorders (e.g., hyperadrenocorticism), inflammatory diseases, clinically significant hypoalbuminemia and anemia, or neurological conditions (e.g., meningoencephalitis of unknown etiology); and (3) administration of glucocorticoids or immunosuppressive agents, which could influence blood parameters, within 30 days prior to the diagnosis of CHF.

### 2.2. HALP Score Calculation

Blood samples were collected from the jugular or cephalic vein of each dog into tubes containing ethylenediaminetetraacetic acid (EDTA) and into serum separator tubes. A complete blood count was performed using an automated hematology analyzer (IDEXX ProCyte Dx; IDEXX Laboratories, Inc., Westbrook, ME, USA), from which hemoglobin, lymphocyte, and platelet counts were obtained. The serum albumin concentration was measured using a biochemical analyzer (Hitachi 7020; Hitachi High-Technologies Co., Tokyo, Japan). The HALP score was calculated using the following formula: Hemoglobin (g/L) × Albumin (g/L) × Lymphocytes (/L)/Platelets (/L). The HALP score for each dog was calculated using laboratory data obtained at the time of CHF diagnosis.

### 2.3. Outcome Assessment

To investigate the clinical significance of the HALP score and its association with poor outcomes in dogs with CHF, poor outcomes were defined as six-month, nine-month, and one-year mortality. Outcomes were assessed at the last follow-up appointment or via telephone interviews with the owners.

### 2.4. Statistical Analysis

All statistical analyses were performed using commercial statistical software (Prism 8; GraphPad Software Inc., La Jolla, CA, USA). A *p* value < 0.05 was considered statistically significant. The normality of continuous variables was assessed using the Shapiro–Wilk test. Normally distributed variables are expressed as mean ± standard deviation, while non-normally distributed variables are expressed as median and interquartile range. The unpaired *t*-test or the Mann–Whitney *U*-test was used to compare continuous variables between the survival and non-survival groups, as appropriate. Categorical variables are expressed as frequencies and percentages, and the Pearson’s chi-square test was used to compare their distribution between groups.

Receiver operating characteristic (ROC) curve analysis was performed to assess the predictive power of the HALP score for six-month, nine-month, and one-year mortality in dogs with CHF. The area under the curve (AUC) was calculated, and diagnostic accuracy was classified based on the AUC value as follows: sufficient (0.6–0.7), good (0.7–0.8), very good (0.8–0.9), and excellent (0.9–1.0). The optimal cut-off value was determined using the highest Youden’s index value (Sensitivity + Specificity − 1).

## 3. Results

### 3.1. Study Population

A total of 54 dogs with CHF secondary to MMVD were included in this study. The demographic characteristics and baseline hematological data for the study population are summarized in Table 1. The cohort consisted of 23 Maltese (42.6%), 11 Shih Tzu (20.4%), 7 Toy or Miniature Poodles (13.0%), 5 Pomeranians (9.3%), 3 Chihuahuas (5.6%), 3 Yorkshire Terriers (5.6%), 3 mixed-breed dogs (5.6%), 2 Spitz (3.7%), and 1 Schnauzer (1.9%).

The 54 dogs were initially stratified into groups based on mortality status at six-, nine-, and twelve-months post-diagnosis. All 54 dogs were included in the six-month mortality analysis. However, eight dogs were excluded from subsequent analyses due to incomplete follow-up data regarding their time of death. Consequently, 46 dogs were assessed for nine-month and one-year mortality outcomes.

The distribution of pre-medicated dogs between the non-survival and survival groups was as follows: 5 and 13 at six months; 8 and 8 at nine months; and 9 and 7 at one year, respectively.

### 3.2. Comparesion of HALP Score

**Figure 1:**
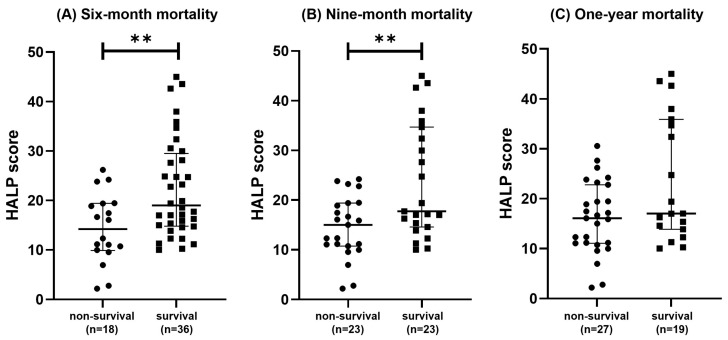
Scatterplots comparing the HALP score between survival and non-survival groups at six, nine, and twelve months in dogs with CHF due to MMVD. The HALP score was significantly higher in the survival group than in the non-survival group at both six months (**A**; *p* = 0.0067) and nine months (**B**; *p* = 0.0080). However, no significant difference was observed at one year (**C**; *p* = 0.0741). The double asterisk (**) indicates a statistically significant difference (*p* < 0.01) determined by the Mann–Whitney *U* test. Horizontal bars indicate the median, and vertical lines represent the interquartile range. CHF, congestive heart failure; HALP, hemoglobin, albumin, lymphocyte, and platelet; MMVD, myxomatous mitral valve disease.

The demographic and clinical data for the survival and non-survival groups at six, nine, and twelve months are presented in Table 2, Table 3, and Table 4, respectively. The HALP score was significantly higher in the survival group compared to the non-survival group at both the six-month (*p* = 0.0067; Figure 1A) and nine-month (*p* = 0.0080; Figure 1B) time points. However, no significant difference in the HALP score was observed between groups for one-year mortality (*p* = 0.0741; Figure 1C).

At the six-month follow-up, the platelet count was significantly higher in the non-survival group (*p* = 0.0133). For the nine-month mortality analysis, both the lymphocyte count (*p* = 0.0048) and the HALP score (*p* = 0.0080) were significantly higher in the survival group. No significant differences in any other clinical variables were observed for the one-year mortality outcome.

### 3.3. ROC Curve of the HALP Score for Short-Term Mortality

**Figure 2:**
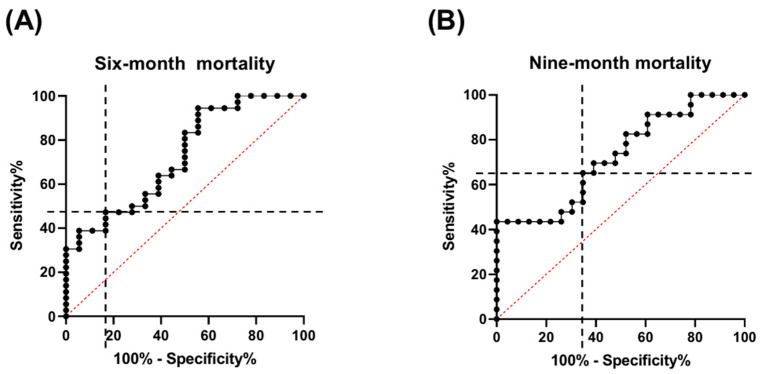
ROC curve analysis of the HALP score for predicting mortality in dogs with CHF due to MMVD at (**A**) six and (**B**) nine months. The AUC was 0.72 (95% CI, 0.58–0.86) for six-month mortality and 0.72 (95% CI, 0.58–0.87) for nine-month mortality. The optimal cut-off value for predicting six-month mortality was 11.13, with a sensitivity of 44.44% (95% CI, 24.56–66.28%) and a specificity of 94.44% (95% CI, 81.86–99.01%). For nine-month mortality, the optimal cut-off was 16.82, with both sensitivity and specificity at 65.22% (95% CI, 44.89–81.19%). The black dashed lines represent the sensitivity and specificity at the optimal cut-off point determined for each graph. AUC, area under the curve; CHF, congestive heart failure; CI, confidence interval; MMVD, myxomatous mitral valve disease; ROC, receiver operating characteristic.

ROC curve analysis was performed to evaluate the predictive accuracy of the HALP score for six-month and nine-month mortality. For six-month mortality (Figure 2A), the AUC was 0.72 (95% confidence interval [CI], 0.58–0.86), with an optimal cut-off value of 11.13. At this cut-off value, the sensitivity was 44.44% (95% CI, 24.56–66.28%) and the specificity was 94.44% (95% CI, 81.86–99.01%).

For nine-month mortality (Figure 2B), the AUC was also 0.72 (95% CI, 0.58–0.87), with an optimal cut-off of 16.82. At this value, both sensitivity and specificity were 65.22% (95% CI, 44.89–81.19%).

### 3.4. Kaplan–Meier Survival Analysis Based on HALP Score

**Figure 3:**
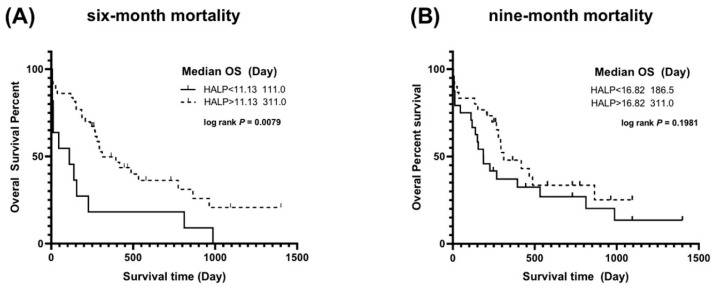
Kaplan–Meier survival curves for dogs with CHF due to MMVD, stratified by the HALP score. The cohorts were divided based on the optimal cut-off values for predicting (**A**) six-month and (**B**) nine-month mortality. (**A**) For the six-month analysis, dogs with a HALP score > 11.13 had a significantly longer median survival time (311 days) compared to those with a score ≤ 11.13 (111 days; *p* = 0.0079). (**B**) For the nine-month analysis, there was no significant difference in median survival time between dogs with a HALP score > 16.82 (311 days) and those with a score ≤ 16.82 (186.5 days; *p* = 0.1981).

Kaplan–Meier survival analysis was performed to compare overall survival between groups categorized by the HALP score cut-off values derived from the six- and nine-month analyses. For the six-month analysis (Figure 3A), dogs with a HALP score >11.13 had a significantly longer median survival time (311 days) compared to dogs with a HALP score ≤ 11.13 (111 days) (*p* = 0.0079). In contrast, for the nine-month analysis (Figure 3B), no significant difference in survival time was observed between dogs with a HALP score >16.82 (median survival time, 311 days) and those with a HALP score ≤ 16.82 (median survival time, 186.5 days) (*p* = 0.1981).

## 4. Discussion

This study evaluated the HALP score as a prognostic indicator for six-, nine-, and twelve-month mortality in dogs diagnosed with CHF due to MMVD. The results indicated that the HALP score was significantly higher in the survival group compared to the non-survival group at both six and nine months. Furthermore, ROC curve analysis identified an optimal cut-off value for the HALP score, and its utility as a short-term prognostic marker was supported by significant differences in six-month mortality. However, its usefulness in predicting nine-month mortality appeared limited. These findings suggest that the HALP score is more effective as a short-term prognostic indicator, particularly for six-month mortality, than as a mid- to long-term survival predictor. The clinical utility of these findings lies in their potential to guide early and targeted interventions to improve patient outcomes.

In human medicine, the HALP score has been extensively studied as a prognostic indicator for various diseases, including cancers, pancreatitis, and heart failure [12,13,14,15]. In gastric cancer, high HALP scores are associated with significantly better survival outcomes compared to low HALP scores, highlighting their value in identifying high-risk patients [17]. Similarly, in pancreatic cancer, low HALP scores have been linked to more aggressive disease features, such as lymph node metastasis and advanced stages, as well as poorer recurrence-free and overall survival post-surgery [18]. The HALP score has also demonstrated excellent prognostic value for short-term mortality in patients with acute pancreatitis. With a high sensitivity (82.8%) and specificity (86.8%) and an AUC of 0.891, the score has proven its reliability as a prognostic indicator in this context [12].

Like its utility in other diseases, the HALP score has shown prognostic value in assessing outcomes for patients with heart failure. In one study, the HALP score was significantly higher in acute decompensated heart failure survivors compared to non-survivors and demonstrated high sensitivity (84%) and specificity (64.1%) in predicting short-term mortality, with an AUC of 0.765 [16]. Another human study showed that the HALP score effectively predicts both short- and long-term mortality in patients with heart failure, with utility comparable to other inflammatory biomarkers such as C-reactive protein, the prognostic nutritional index, and the Meta-Analysis Global Group in Chronic Heart Failure risk score [15]. Thus, the HALP score has been validated as a reliable predictor of short-term mortality in human heart failure.

Consistent with these findings in human medicine, our study highlights the utility of the HALP score in predicting six-month mortality in dogs with CHF due to MMVD. The pathogenesis of CHF is closely linked to cell-mediated immune responses, inflammation, and oxidative stress—all of which play critical roles in disease progression and severity [19,20,21]. The four components of the HALP score—hemoglobin, albumin, lymphocyte count, and platelet count—each serve as key indicators reflecting these underlying pathophysiological processes [16,22,23].

CHF is a state of chronic inflammation that contributes to anemia through suppressed erythropoiesis. Elevated levels of pro-inflammatory cytokines, such as tumor necrosis factor-alpha (TNF-α), interleukin (IL)-6, and IL-1, inhibit erythropoietin production in the kidneys and suppress erythroid progenitor cell proliferation in the bone marrow [24,25]. Additionally, IL-6 stimulates hepcidin production, which reduces iron absorption and restricts the release of stored iron, leading to functional iron deficiency and impaired hemoglobin synthesis. These processes ultimately reduce tissue oxygenation, worsening myocardial oxygen demand and impairing cardiac function, which negatively impacts survival [25,26].

Similarly, albumin levels, an indicator of nutritional status, often decrease in CHF due to malnutrition and chronic inflammation. Pro-inflammatory cytokines like IL-6 promote the production of acute-phase proteins while suppressing albumin synthesis, leading to hypoalbuminemia [27]. Hypoalbuminemia can decrease colloid osmotic pressure and exacerbate pulmonary congestion, ultimately contributing to a poor prognosis [28].

Lymphopenia is a significant marker of immune dysregulation and systemic inflammation in CHF. It is driven by neurohumoral activation, with elevated cortisol and catecholamine levels suppressing lymphocyte proliferation [29]. In CHF, lymphopenia may also be exacerbated by elevated levels of circulating endotoxins, which are commonly observed in severe heart failure due to gut translocation from congestion and hypoperfusion [30,31]. This heightened inflammatory state contributes to lymphocyte apoptosis and suppresses their production [32].

The inflammatory state associated with CHF also increases platelet production and promotes platelet aggregation as part of the systemic response [33]. IL-6 and TNF-α further activate platelets, contributing to vascular dysfunction and thrombosis [34,35]. Consequently, thrombocytosis can impair tissue perfusion, worsen the clinical severity of CHF, and negatively affect survival outcomes [33].

In veterinary medicine, prognostic indicators for CHF remain limited, highlighting the clinical importance of our findings. To date, studies have primarily focused on identifying markers that assess the severity of MMVD or predict its progression to CHF [2,3,4,5,6,7,8,9,10]. We found that lower HALP scores were significantly associated with poorer short-term survival in dogs with CHF secondary to MMVD. This finding is in line with the concept that inflammation and malnutrition contribute to adverse outcomes in heart failure. Specifically, hypoalbuminemia observed in non-survivors may reflect both chronic inflammation and impaired nutritional status, while lymphopenia indicates immune dysregulation frequently reported in CHF. In addition, the elevated platelet counts in the non-survival group are consistent with the pro-thrombotic state driven by inflammatory cytokines. Taken together, the present results provide direct evidence that the prognostic impact of the HALP score in our canine cohort is closely related to the underlying inflammatory and nutritional mechanisms previously described in human heart failure. Thus, this correlation supports the clinical relevance of HALP as an integrative marker linking systemic inflammation, nutritional depletion, and survival in dogs with CHF due to MMVD. Furthermore, the study highlights the HALP score’s clinical utility, supported by its strong accuracy and high specificity for predicting six-month mortality. Given the scarcity of effective survival predictors in veterinary medicine, these results suggest the clinical relevance of the HALP score as a valuable tool that reflects a patient’s nutritional and inflammatory status, thereby enhancing prognostic assessment and guiding clinical decisions.

Some dogs with CHF secondary to MMVD died early while others survived longer due to multiple factors influencing disease progression and individual variation. Differences in systemic inflammation, nutritional status, immune dysregulation, and severity of cardiac dysfunction likely contributed. In our study, lower HALP scores—reflecting anemia, hypoalbuminemia, lymphopenia, and platelet alterations associated with chronic inflammation and malnutrition—were significantly linked to early mortality. This suggests that dogs with more severe inflammatory and nutritional impairment had poorer outcomes. Additionally, variability in treatment response, comorbidities, and other unmeasured factors may have influenced survival times. Further research with larger cohorts and detailed clinical parameters is needed to fully understand the complex reasons behind different survival outcomes.

The criteria for a “good” HALP score vary depending on the disease, but generally, a higher score is considered indicative of a better prognosis as it reflects a more favorable immune and nutritional status. However, the specific cut-off value that defines a “good” score differs by study and disease type [36]. For instance, in gastric cancer, the HALP cutoff ranges approximately from 35 to 57, whereas colorectal cancer studies often report lower cutoff values between 15 and 32. Lung cancer studies demonstrate a broader range from about 18 to 48, while bladder and urothelial cancers generally show cutoffs between 22 and 31. This wide heterogeneity emphasizes that the HALP score cutoff should be tailored specifically to each cancer subtype and study context. Consequently, there is no universal threshold applicable across all malignancies, highlighting the need for disease- and outcome-specific optimization when utilizing HALP as a prognostic biomarker.

The HALP score is a strong predictor of short-term mortality because it integrates key indicators of a patient’s nutritional status (albumin), oxygen-carrying capacity (hemoglobin), immune function (lymphocytes), and inflammatory/coagulation response (platelets). These combined factors reflect the overall health and resilience of the patient, which are critically important in determining survival during acute illness or stress. This multifaceted biomarker thus provides a comprehensive assessment of physiological vulnerability, making it highly effective for early identification of patients at risk of death in the short term [12,13,14,15,16]. Therefore, the HALP score is widely utilized as a clinical indicator to quickly assess short-term prognosis.

Despite these promising results, this study has several limitations. The relatively small sample size may limit the generalizability of the findings; therefore, larger, multi-center studies are needed to validate the HALP score’s utility as a prognostic marker. In stage B2, which precedes the onset of CHF, medications such as pimobendan or ACE inhibitors are commonly prescribed. Anemia is a key side effect of ACE inhibitors, developing through two primary mechanisms [37]. First, they cause a dose-dependent suppression of erythropoietin, which is particularly problematic in patients with renal failure. Second, they lead to the accumulation of N-acetyl-seryl-aspartyl-lysyl-proline, a potent natural inhibitor of hematopoietic stem cell proliferation that is normally degraded by ACE. While the effects of pimobendan on hemoglobin, albumin, lymphocytes, and platelets are not well-documented, it is plausible that the HALP score was influenced by the effects of ACE inhibitors. Additionally, variability in post-pulmonary edema treatment among the dogs may have influenced individual prognoses, potentially affecting the consistency of the score’s predictive power.

## 5. Conclusions

This study suggests that the HALP score is a valuable and practical prognostic tool for predicting short-term mortality in dogs with CHF caused by MMVD. The score provides critical information for both veterinarians and pet owners, enabling the early identification of high-risk patients and guiding more intensive management strategies. Its simplicity, non-invasive nature, and cost-effectiveness make it an asset in clinical decision-making and treatment planning. Furthermore, a clearer understanding of their pet’s prognosis empowers owners to make more informed treatment decisions. Future studies should focus on validating the HALP score in larger, more diverse populations and exploring its broader application to other chronic and inflammatory diseases in veterinary medicine.

## Figures and Tables

**Table 1:** Demographic characteristics of dogs with CHF due to MMVD.

	Dogs with CHF (*n* = 54)
Age (years)	13.79 ± 3.43
Sex	
Male	26 (48.1%)
Female	28 (51.9%)
Breeds	
Maltese	23 (39.7%)
Shih Tzu	11 (20.3%)
Miniature or Toy Poodle	7 (12.9%)
Pomeranian	5 (7.4%)
Chihuahua	3 (5.5%)
Yorkshire Terrier	3 (5.5%)
Mixed breeds	3 (5.5%)
Spitz	2 (3.7%)
Miniature Schnauzer	1 (1.8%)

CHF, congestive heart failure; MMVD, myxomatous mitral valve disease.

**Table 2:** Comparison of demographic and clinical data according to six-month mortality.

		Non-Survival (*n* = 18)	Survival (*n* = 36)	*p*-Value
Age (years)		12.5 (9.9–14.0)	12.5 (10.6–14.7)	0.6725
Body weight (kg)		3.08 (2.61–4.52)	3.76 (2.95–5.07)	0.2442
Sex (number)	Male	8	18	0.7773
	Female	10	18	
Hemoglobin (g/L) (RI, 131–205)		151.7 ± 23.41	157.9 ± 26.46	0.3953
Albumin (g/L) (RI, 26–33)		25.50 (22.75–36.50)	28.00 (25.00–32.00)	0.9239
Lymphocyte (10^9^/L) (RI, 1.05–5.10)		1.485 (1.265–1.788)	1.745 (1.206–2.470)	0.1206
Platelet (10^9^/L) (RI, 148–484)		467.0 ± 145.7	378.8 ± 106.8	0.0133 *
Neutrophil (10^9^/L) (RI, 2.95–11.64)		10.31 (8.048–12.41)	8.280 (6.536–10.70)	0.1618
HALP score		14.20 (9.883–19.41)	18.99 (14.78–29.50)	0.0067 **

Continuous data with a normal distribution were presented as the mean ± standard deviation and compared between groups using an unpaired *t*-test. Non-normally distributed data were presented as the median (interquartile range) and compared between groups using the Mann–Whitney *U* test. Categorical data were presented as counts (percentages) and analyzed using the Pearson’s Chi-square test or Fisher’s exact test, as appropriate. A *p*-value < 0.05 was considered statistically significant. * Statistically significant at *p* < 0.05. ** Statistically significant at *p* < 0.01. HALP, hemoglobin, albumin, lymphocyte, and platelet; RI, reference interval.

**Table 3:** Comparison of demographic and clinical data according to nine-month mortality.

		Non-Survival (*n* = 23)	Survival (*n* = 23)	*p*-Value
Age (years)		13.00 (9.723–14.44)	12.79 (10.76–14.11)	0.7892
Body weight (kg)		4.06 (2.98–5.72)	3.34(2.93–5.07)	0.3417
Sex (number)	Male	10	11	0.7672
	Female	13	12	
Hemoglobin (g/L) (RI, 131–205)		151.8 ± 23.48	158.0 ± 28.57	0.4273
Albumin (g/L) (RI, 26–33)		27.00 (23.00–36.00)	28.00 (25.00–31.20)	0.9176
Lymphocyte (10^9^/L) (RI, 1.05–5.10)		1.370 (0.9890–1.700)	1.870 (1.560–2.410)	0.0048 **
Platelet (10^9^/L) (RI, 148–484)		406.0 (348.0–492.0)	373.0 (270.0–486.0)	0.2358
Neutrophil (10^9^/L) (RI, 2.95–11.64)		9.950 (5.580–12.39)	8.953 (7.170–10.79)	0.9826
HALP score		14.98 (10.72–19.40)	17.73 (14.55–34.67)	0.0080 **

Continuous data with a normal distribution were presented as the mean ± standard deviation and compared between groups using an unpaired *t*-test. Non-normally distributed data were presented as the median (interquartile range) and compared between groups using the Mann–Whitney *U* test. Categorical data were reported as counts (percentages) and analyzed using the Pearson’s chi-square test or Fisher’s exact test, as appropriate. Statistical significance was defined as *p* < 0.05. ** Statistically significant at *p* < 0.01. HALP, hemoglobin, albumin, lymphocyte, and platelet; RI, reference interval.

**Table 4:** Comparison of demographic and clinical data according to one-year mortality.

		Non-Survival (*n* = 27)	Survival (*n* = 19)	*p*-Value
Age (years)		12.80 (11.00–14.93)	11.52 (9.96–13.4)	0.0829
Body weight (kg)		3.06 (2.50–4.80)	3.80(2.96–7.00)	0.1148
Sex (number)	Male	12	8	0.9035
	Female	15	11	
Hemoglobin (g/L) (RI, 131–205)		151.8 ± 23.01	161.6 ± 30.01	0.2156
Albumin (g/L) (RI, 26–33)		29.00 (23.00–36.00)	38.50 (25.00–31.00)	0.3546
Lymphocyte (10^9^/L) (RI, 1.05–5.10)		1.410 (1.100–1.980)	1.760 (1.281–1.950)	0.1404
Platelet (10^9^/L) (RI, 148–484)		447.4 ± 136	374.4 ± 115.4	0.0633
Neutrophil (10^9^/L) (RI, 2.95–11.64)		9.160 (6.290–12.1100)	8.440 (7.010–10.06)	0.7572
HALP score		16.08 (11.06–22.78)	17.01 (13.86–35.9)	0.0741

Continuous data with a normal distribution were presented as the mean ± standard deviation and compared between groups using an unpaired *t*-test. Non-normally distributed data were presented as the median (interquartile range) and compared between groups using the Mann–Whitney *U* test. Categorical data were presented as counts (percentages) and analyzed using Pearson’s chi-square test or Fisher’s exact test, as appropriate. Statistical significance was defined as *p* < 0.05. HALP, hemoglobin, albumin, lymphocyte, and platelet; RI, reference interval.

## Data Availability

The data that support the findings of this study are available on reasonable request from the corresponding author.
